# Central injection of abscisic acid attenuates mood disorders induced by subchronic stress in male mice

**DOI:** 10.1002/brb3.2796

**Published:** 2022-11-10

**Authors:** Mohammad Shabani, Hoda Ranjbar, Monavareh Soti, Reyhaneh Naderi

**Affiliations:** ^1^ Neuroscience Research Center Neuropharmacology Institute Kerman University of Medical Sciences Kerman Iran

**Keywords:** abscisic acid, anxiety, cognitive impairments, depression, swim stress

## Abstract

Stressful life increases the risk of mental and psychological disorders and cognitive deficits. Abscisic acid (ABA) is a plant hormone that has been recently discovered in mammalians. ABA is produced in response to stressful stimuli and it can reduce anxiety‐like behaviors and depression and improve cognitive function. This study was designed to evaluate the effects of microinjection of ABA on depression, anxiety, passive avoidance learning and memory deficits induced by subchronic stress. ABA (10 and 15 μg/mouse, i.c.v.) was administered one week after recovery period for 4 consecutive days. A three‐session forced swimming test (FST) protocol for induction of subchronic stress was administered to the mice. Exploratory, anxiety‐like behavior, depression and cognitive function were assessed 24 h after the last swim stress session. The results indicated that ABA (15 μg/mouse) could ameliorate anxiety and depression induced by FST. In addition, ABA had no effect on the subchronic stress‐induced cognitive impairments. Taken together, the results suggest that ABA could improve anxiety and depression induced by subchronic stress.

## 1 INTRODUCTION

Stress is a process that interrupts the balance between an organism and its environment and causes physiological, psychological and biochemical changes and affects the functions of body organs (McEwen et al., 2015; Ohl & Fuchs, 1999). Chronic stress (severe and prolonged stress) leads to enhancement of proinflammatory cytokines and oxidative stress markers in the CNS, which has adverse effects and increases risk for development of diseases (Kubera et al., 2011; Maes, 1993). Exposure to uncontrollable stressful situations can lead to mental disorders including depression and anxiety (Khan & Khan, 2017; McEwen, 2004; Ohl & Fuchs, 1999). In addition, chronic stress has been demonstrated to change cognitive functions (Maeng & Shors, 2013).

Swimming can be considered chronic stress for rodents in their natural environment. Rodents are exposed to psychological stress (fear of drowning) and physical stress (swimming) (Suarez‐Roca et al., 2014). Forced swim test (FST) has been introduced by Quintero and colleagues as an animal model of subchronic stress. In this model, animals are forced to swim in a cylinder from which there is no escape (Quintero et al., 2000a).

Abscisic acid (ABA) is a plant hormone that mediates several important functions in plants including plant growth and development, regulation of seed dormancy and senescence, control of stomatal closure, and responses to environmental stresses (Cutler et al., 2010; Kim et al., 2010). Recently, it has been reported that this phytohormone also exists in animals and it can act as a stress signal, which regulates different cell functions (Bodrato et al., 2009; Bruzzone et al., 2007; Magnone et al., 2009; Magnone et al., 2012). ABA is produced in response to environmental stimuli in lower Metazoa and temperature rise in sponges or light exposure in hydroids has been shown to stimulate water filtration and tissue regeneration, respectively (Puce et al., 2004; Zocchi et al., 2003, 2001). It has been indicated that ABA activates immune cells and regulates inflammatory processes (Bodrato et al., 2009; Bruzzone et al., 2007; Magnone et al., 2012) and glucose homeostasis in mammals (Bruzzone et al., 2008, 2012; Guri et al., 2007).

Although ABA is obtained through nutrition (Magnone et al., 2015), it can also produce in different tissues of the animal's body (Bodrato et al., 2009; Bruzzone et al., 2007; Le Page‐Degivry et al., 1986; Magnone et al., 2009, 2012; Scarfì et al., 2008). Previous studies have demonstrated that ABA plays a neurophysiological role in mammals and it exists in various brain areas including hypothalamus, hippocampus, cortex, and cerebellum (Qi, Ge & Zhou, 2015a; Qi et al., 2015b). ABA can improve cognitive function in intact rats and rats with diseases such as Alzheimer's (Khorasani, Abbasnejad & Esmaeili‐Mahani, 2019), tremor (Shabani & Naderi, 2022), and diabetes (Kooshki et al., 2021). In addition, this substance has been indicated to attenuate motor disturbances in in vitro model of Parkinson's disease (PD) (Rafiepour et al., 2019). It has been reported that ABA is involved in mood state, which reduces anxiety‐like behaviors (Naderi, Esmaeili‐Mahani & Abbasnejad, 2017) and depression (Qi et al., 2015b).

Previously, it has been reported that ABA can improve cognitive performance and inhibit depression and anxiety‐like behaviors (Naderi et al., 2017; Naderi, Esmaeili‐Mahani & Abbasnejad, 2019; Qi et al., 2015a). Since the effect of ABA on the experimental model of subchronic stress has not yet been reported, the present study was designed to find the role microinjection of ABA on depression, anxiety, and passive avoidance learning and memory deficits induced by FST in mice.

## 2 MATERIALS AND METHODS

### 2.1 Animals

Male Swiss mice weighing 25–35 g were purchased from the Kerman University of Medical Sciences. The mice were housed under a 12 h light/dark cycle in controlled condition with temperature of 22 ± 2°C, and ad libitum access to food and water. The Kerman University of Medical Sciences Ethics Committee (IR.KMU.REC.1400.042) approved all the procedures.

### 2.2 Experimental design

**FIGURE 1:**
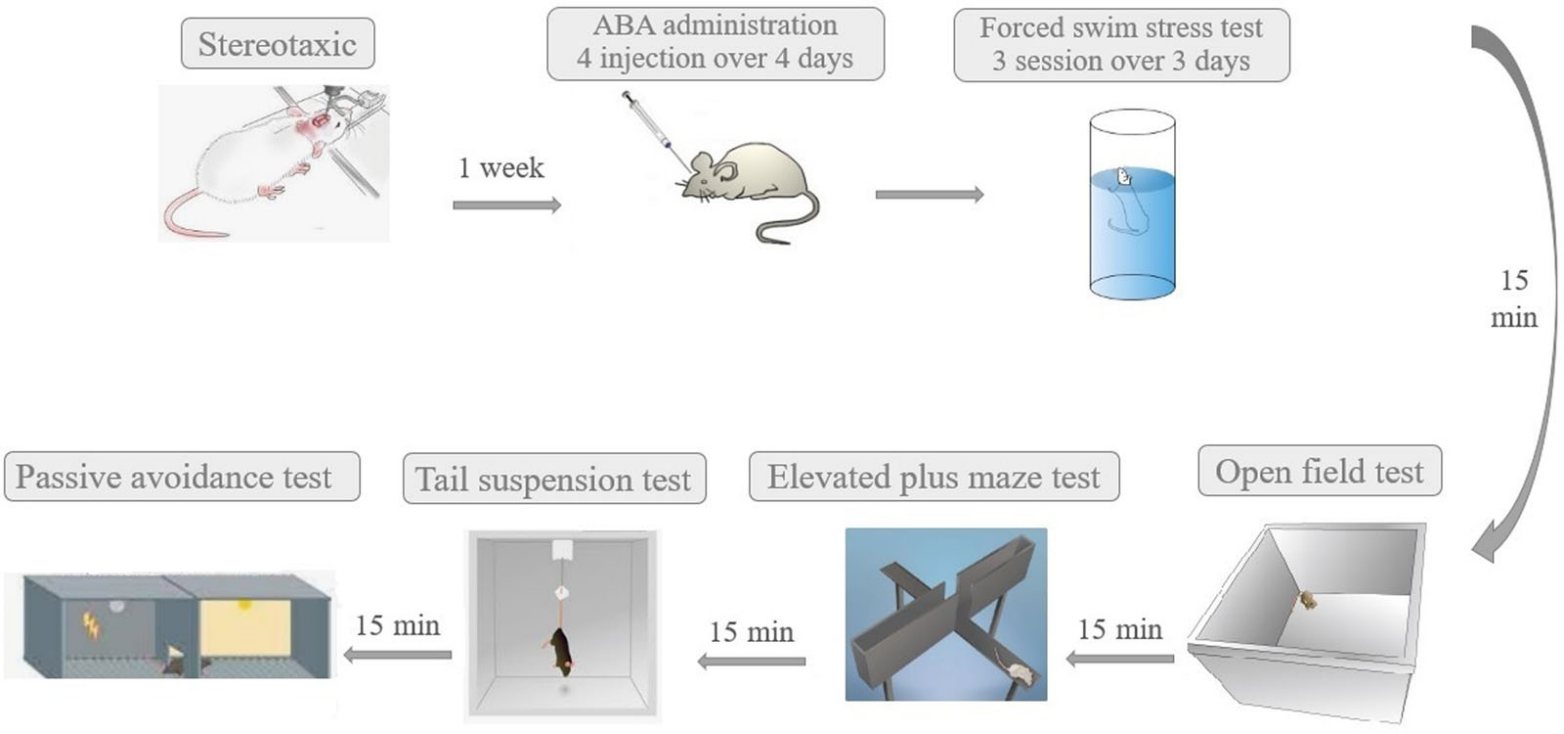
The experimental design and study timeline

A brief experimental design timeline is depicted in Figure 1. ABA was administered one week after recovery period for 4 consecutive days. A three‐session FST protocol to induction of subchronic stress was administered to the mice. Behavioral testes were assessed 24 h after the last swim stress session with sequentially 15‐min rest intervals among each assay in the following order: open‐field test, elevated plus maze, tail suspension test, and passive avoidance task. The male mice (*n* = 8/group) were randomly divided into fallowing groups: control group (Cont), which had no surgery and treatment; Stress group (Stress), which underwent 3 days FST; Sham‐operated group (Sham), which received ABA vehicle; ABA‐treated group (ABA), which received 10 μg/mouse ABA before swimming session; dimethyl sulfoxide (DMSO)‐treated stress (Stress + DMSO) group, which underwent 3 days FST and received DMSO; ABA‐treated stress group (Stress + ABA), which received ABA at dose of 10 and 15 μg/mouse before swimming session. Due to the fact that it is a relatively new hormone found in mammals in the past few years, there have not been many studies conducted in this area. Using dose–response and effective dose selection data from our previous lab studies, the selected dose was chosen.

### 2.3 Surgery and microinjection

In order to intracerebroventricular (i.c.v.) injection of drugs, the animals were anesthetized with intraperitoneal injection a mixture of ketamine and xylazine (90 and 10 mg/kg, respectively). Guide cannulas were implanted bilaterally at the coordinates of: 0.3 mm posterior to the bregma, ±1.0 mm lateral from the midline, and 2.5 mm depth to the cortical surface above the lateral ventricles (Paxinos et al., 1980). The implanted cannula was fixed to the skull surface by two screws and dental cement. After surgery, the animals were transferred to their home cages and given 1‐week recovery week before behavioral experiments. After recovery period, (±)‐cis, trans‐ABA (Sigma‐Aldrich, USA) was dissolved in the sterile saline solution (0.9% w/v sodium chloride) with DMSO at a ratio of 2:1 (v/v) and delivered into cerebral lateral ventricles for four consecutive days. A 27‐gauge needle connected by a polyethylene tube to a Hamilton syringe was used for the drugs injection (intracerebroventricularly, i.c.v.). The injection needle was remained in the place for additional 1 min to ensure a complete diffusion of the drugs.

### 2.4 Forced swimming test (FST)

It has already been shown that FST elicits stress responses (Quintero et al., 2000b). The FST paradigm was used to induction of subchronic stress and performed on 3 days. Mice separated and were forced to swim in a cylinder (50 cm height × 50 cm diameter) containing 20 cm of water (24 ± 1°C) for a period of 5 min on three consecutive days. After swimming sessions, the animals were removed from the cylinders, carefully dried with towels, and then returned to their home cages (Mohammad‐Zadeh et al., 2014; Petit‐Demouliere et al., 2005).

### 2.5 Open‐field test

The open‐field apparatus consisted of a square arena (90 × 90 × 45 [H] cm), which floor was marked with a grid dividing the floor into 16 equal‐size squares. Squares adjacent to the walls were considered as the peripheral zone, whereas the remaining squares were considered as the central zone. The animals were placed in the center arena and their activities were recorded for 5 min and assayed using Ethovision software (Ethovision7.1, Noldus Information Technology, the Netherlands). The apparatus was cleaned with 75% ethanol before each test. Total distance moved, velocity, grooming, time spent in the center of the open field, and total locomotor activity were measured (Mohammadi et al., 2019).

### 2.6 Elevated plus maze test

The maze was conducted on a plus‐shaped apparatus that was elevated 50 cm above the floor and consisted of two open arms (50 cm) and two closed arms (50 cm) connected with a central square (10 × 10 cm). The mice were placed gently in the center of the device facing the open arm and allowed to explore the maze for 5 min. The percent of time and number of entries into the open was recorded manually. An increase in open arm duration/entries reflects antianxiety behavior. The Apparatus was cleaned with cotton and 70% ethanol between each test (Nazeri et al., 2015).

### 2.7 Passive avoidance test

A shuttle‐box apparatus was used to analysis associative learning and memory. The maze consisted of two parts (a light and a dark chamber) with a sliding guillotine door. Each animal was first habituated to the test by placement in the light chamber (door closed) for 10 s. Then the door was opened and the animal was allowed to enter the dark chamber and was explored for 30 s. In the learning trial, each mouse was located in light chamber of the apparatus and then the door was opened the mouse was allowed to enter into the dark cavity. Then, the gate was closed and an electric shock (0.5 mA, 50 Hz) delivered to the animal for 2 s. This last step was repeated at 30 min intervals until the animal learned to avoid the dark chamber. The number of learning trials to get efficient learning was recorded. After 30 min, the same test was conducted again, and if the mice did not enter the dark chamber by 300 s, the successful acquisition of passive avoidance response was recorded (shock number) and the number of allowable shocks was 5. Twenty‐four hours after learning phase, the retention trial was performed to assess memory. In this step, the animals were placed in the light compartment of the apparatus. After 10 s, the gate was opened and the time before the first entry of the animal to the dark chamber was considered as step‐through latency (STL) during a 300 s interval (Nazeri et al., 2014a).

### 2.8 Statistical analysis

Normally distributed data were compared by a one‐way ANOVA and the Tukey's post hoc analysis was used for multiple comparisons between other groups. Nonnormally distributed data were analyzed using a Kruskal–Wallis test. Where a main effect was seen, pairwise comparisons between groups were then made using Dunn's multiple comparisons test. Data are expressed as mean ± SEM and *p* < .05 was considered statistically significant between groups.

## 3 RESULTS

### 3.1 Effect of swim stress and pretreatment with ABA on explorative and anxiety‐related behaviors in open field

**FIGURE 2:**
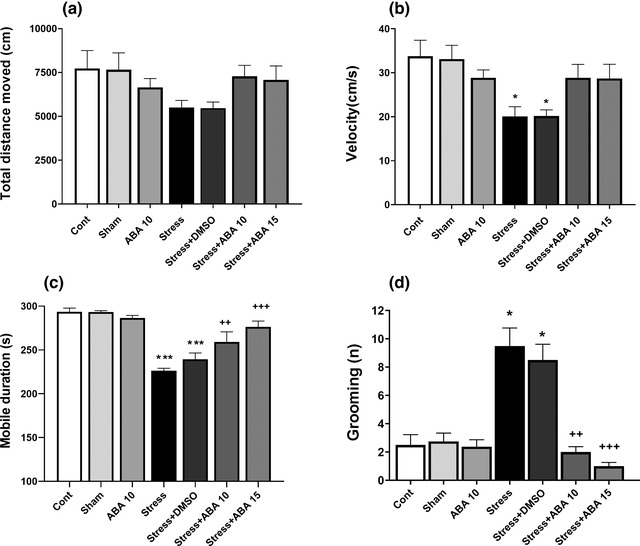
Effect of ABA (10 and 15 μg/rat) on explorative and anxiety like behavioral changes induced by swim stress. Total distance moved (a), velocity (b) and mobility duration (c), and grooming number (d). Data presented as mean ± SEM. *p < .05 and *p < .001 versus control group; ^++^ *p* < .01 and ^+++^ *p* < .01 versus stress group

As shown in Figure 2, there was no significant difference between groups in total distance moved (*F*(6, 49) = 1.784, *p* = .1219). Moreover, Stress and Stress+DMSO groups significantly showed decreased velocity (*p* < .05) and mobility duration (*p* < .001) compared to the control group. ABA in doses of 10 (*p* < .01) and 15 μg/mouse (*p* < .001) reversed mobility duration compared to the control level. In addition, FST significantly increased the number of grooming in the Stress and Stress+DMSO groups as compared to the control group (*p* < .05). The administration of ABA (10 μg/mouse) (*p* < .01) and (15 μg/mouse) (*p* < .001) could restore increased grooming induced by swim stress.

### 3.2 Effect of swim stress and pretreatment with ABA on anxiety‐like behaviors in elevated plus maze

**FIGURE 3:**
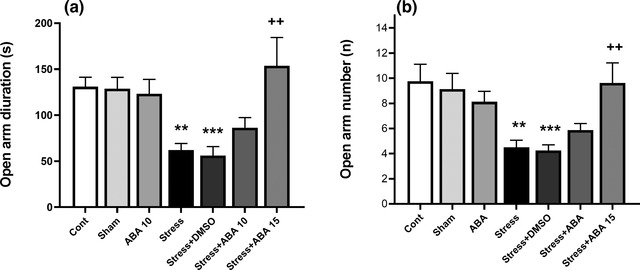
Effect of ABA (10 and 15 μg/rat) on anxiety like behavioral changes induced by swim stress. Open arm duration (a) and (b) open arm number. Data presented as mean ± SEM. **p < .01 and ***p < .001 versus control group; ^++^ *p* < .01 versus stress group

As illustrated in Figure 3, there were significant differences in time spent (*F*(6, 49) = 11.24, *p* = .0001) and number of entries (*F*(6, 49) = 7.700, *p* = .0001) in the open arms between experimental groups. Mice in the Stress (*p* < .01) and Stress+DMSO (*p* < .001) groups had a decreased time spent and number of entries in the open arms as compared to the control group. The microinjection of ABA (15 μg/mouse) significantly increased the time spent and number of entries in open arms (*p* < .01) as compared to the control animals (Figure 3a and b).

### 3.3 Effect of swim stress and pretreatment with ABA on depression in tail suspension

**FIGURE 4:**
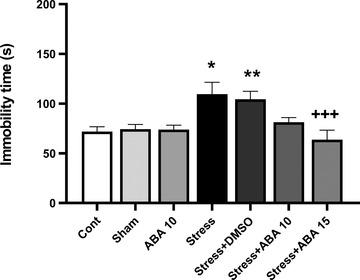
Effect of ABA (10 and 15 μg/rat) on immobility time changes induced by swim stress. Data presented as mean ± SEM. *p < .05 and **p < .01 versus control group; ^+++^ *p* < .001 versus stress group

There was a significant difference (*F*(6, 49) = 128.3, *p* = .0001) in immobility time between groups in the tail suspension test (Figure 4). Swim stress significantly increased immobility time in Stress (*p* < .05) and Stress+DMSO (*p* < .01) groups in comparison with control animals. The pretreatment with 15 μg/mouse ABA (*p* < .001) was able to prevent the anti‐immobility effect induced by FST.

### 3.4 Effect of swim stress and pretreatment with ABA on learning and memory in shuttle box

**FIGURE 5:**
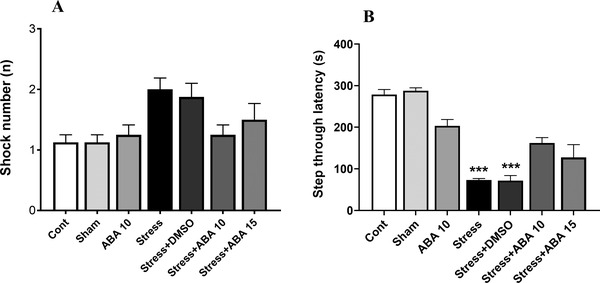
Effect of ABA (10 and 15 μg/rat) on learning and memory changes induced by swim stress after harmaline administration. Number of shock (a), step through latency (STL) (b). ***p < .001 versus control group

As shown in Figure 5a, there was no significant difference between experimental groups in the number of received shocks (*F*(6, 49) = 3.725, *p* = .0039). In addition, the results indicated that STL significantly decreased in Stress and Stress+DMSO groups as compared to the control animals (*p* < .001). However, treatment with ABA did not change cognitive impairment induced by FST (Figure 5b).

## 4 DISCUSSION

In this study, our results have indicated that chronic FST causes anxiety‐like behaviors, and cognitive dysfunction in mice. As optimized, i.c.v administration of ABA ameliorated anxiety and depression in a subchronic stress model. In addition, pretreatment with ABA could not attenuate passive avoidance memory deficits induced by subchronic stress.

Unpredictable and uncontrollable stressful conditions lead to neurochemical, neurotransmitter, and hormonal changes in central nervous system to help the organism reacts appropriately for adaptation with challenging situation. Repeated life stress is associated with neuropsychiatric disorders such as depression and anxiety, and type and duration of stress affect the development of these mental diseases (Khan & Khan, 2017; McEwen, 2004; Ohl & Fuchs, 1999) .In addition, numerous studies have emphasized the cognitive deficits caused by stress (Lupien et al., 2007; Maeng & Shors, 2013; Radecki et al., 2005; Wright & Conrad, 2008). Previous studies have been indicated that swim stress increased anxiety‐like behavior and impairs passive avoidance learning and memory (Nazeri et al., 2014b; Nazeri et al., 2018; Ranjbar et al., 2018).

It has been shown that exposure to stress causes excessive release of proinflammatory cytokines, which damage the brain through affecting the metabolism of neurotransmitter, decreasing neurogenesis, neural plasticity, oxidative stress, induction of apoptosis, and stimulating of glutamatergic system (Anisman, Merali & Hayley, 2008; Felger et al., 2007; Kubera et al., 2011).

It has been shown that stress increases production of free radicals and release of inflammatory factors in brain (Luine et al., 1994). Oxidative stress is associated with neuroinflammation, which both are major causes of neurodegenerative disorders (Gelders et al., 2018; McManus & Heneka, 2017; Yaribeygi et al., 2018). Inflammation and oxidative stress have been considered to involve in several behavioral disorders including anxiety, depression, schizophrenia, and cognitive impairments (Balmus et al., 2016; Maes et al., 2011; Uttara et al., 2009; Wu, Kosten & Zhang, 2013).

Phytohormone ABA regulates important physiological processes of plants especially responses to environmental stresses (low temperature, drought, salinity, and pathogens) (Cutler et al., 2010; Kim et al., 2010). It has also been reported in lower species, which acts as an antistress hormone. For instance, temperature rise in sponges triggers the temperature‐signaling cascade result in water filtration and respiration (Zocchi et al., 2003; Zocchi et al., 2001) and mediates tissue regeneration in response to light stimulator in hydroids (Puce et al., 2004).

Recent studies have demonstrated that ABA is produced and accumulated in animal tissues (Bodrato et al., 2009; Bruzzone et al., 2007; Magnone et al., 2009; Magnone et al., 2012; Scarfì et al., 2008). Le Page‐Degivry et al. (1986) reported that ABA concentration is higher in the brain than in other tissues and it can release from different areas of the brain such as hypothalamus, hippocampus, cortex, and cerebellum (Qi et al., 2015a; Qi et al., 2015b). Due to the recent discovery of this hormone in animals, a definite mechanism for its action has not been established, but a few possible mechanisms are stated below.

In addition, it has been shown that ABA is released from innate immune cells and acts as an anti‐inflammatory agent in defensive responses. Physical (temperature rise, latex beads) or chemical (phorbol myristate acetate) stimuli induce ABA production and release in human granulocytes, where ABA activates inflammatory including phagocytosis, cell migration, and production of reactive oxygen species and nitric oxide (Bruzzone et al., 2007). ABA reduced microglial activation and tumor necrosis factor (TNF‐α) production in the hypothalamus in a model of neuroinflammation induced by high‐fat diet. In addition, ABA could reduce inflammation in mouse model of inflammatory bowel disease, decrease pulmonary inflammation in mouse model of influenza virus–associated disease, and ameliorate glucose tolerance and obesity‐related inflammation in db/db mice model.

ABA protects plants against oxidative damage through induction of the antioxidant encoding genes expression and the increasing of antioxidant enzymes activities (Jiang & Zhang, 2004; Sandhu et al., 2011). Moreover, it has been demonstrated that ABA could act as antioxidant agents in various tissues in rats (Celik, Turker & Tuluce, 2007). Central injection of ABA has ROS scavengers’ activity by enhancement of the levels of catalase and peroxidase in diencephalon (Soti et al., 2019).

It was found that forced exercise causes stress responses (Contarteze et al., 2008; Svensson et al., 2016) and inflammation (Cook et al., 2013; Nambot et al., 2020) and damages the brain can lead to behavioral disorders, impair memory (de Quervain, Schwabe & Roozendaal, 2017) and anxiety (Gold, Machado‐Vieira & Pavlatou, 2015). Because oxidative damage and inflammation are involved in neuropsychiatric disorders (Gelders et al., 2018; McManus & Heneka, 2017; Yaribeygi et al., 2018), antioxidants may be able to reduce the symptoms of such disorders. For example, curcumin ameliorates spatial working memory and anxiety‐like behavior through reduction of prefrontal cortex neuroinflammation and oxidative stress (Namgyal et al., 2021). In addition, it has been reported that gallic acid improves anxiety and locomotor activity and memory deficits induced by chronic stress (Dhingra et al., 2014). It is therefore possible that abscisic's antioxidant properties may help reduce anxiety caused by stress.

Main mediators of the stress response regulate peroxisome proliferator and activated receptor gamma (PPARγ) activity in rat brain cortex after acute restraint stress exposure. PPARγ has been shown to have anti‐inflammatory activities in brain through decrease proinflammatory cytokines release or interfere with inflammatory transcription pathways. PPARγ could reduce damages in animal models of inflammatory‐related neurodegenerative diseases (García‐Bueno et al., 2008).

It has been shown that ABA is involved in inflammation and innate immune responses through activation of lanthionine synthetase C‐like protein 2 (LANCL2)/PPARγ, which is known to regulate the inflammatory responses (Baliño et al., 2019). Guri et al. has reported that dietary ABA supplementation ameliorates colonic inflammatory lesions through T‐cell PPARγ‐dependent mechanism (Guri et al., 2011). In addition, ABA improves insulin sensitivity and obesity‐related inflammation through a mechanism requiring immune cell PPARγ (Guri et al., 2010).

In addition, Rafiepour and colleagues have reported that antioxidant properties of ABA mediated via the PPAR signaling cascade in in vitro model of Parkinson's disease. It has been shown that PPARγ agonists have protective effects against oxidative damage (Rafiepour et al., 2019). Other possibilities include PPAR signaling and reducing inflammation as explanations of ABA's observed effects.

In conclusion, the findings of this study confirmed that subchronic swim stress causes anxiety‐like behaviors and cognitive dysfunction. These data showed that ABA attenuates anxiety and depression in a subchronic stress model. Furthermore, central administration of ABA could not improve passive avoidance memory deficits induced by swim chronic stress.

## FUNDING

This study was financially supported by fund from Kerman Neuroscience Research Center, Kerman, Iran.

## CONFLICT OF INTEREST

The authors declare no conflict of interest.

### PEER REVIEW

The peer review history for this article is available at https://publons.com/publon/10.1002/brb3.2796.

## Data Availability

Data will be made available in request.
